# Effects of *Escherichia coli* strain Nissle 1917 on exercise-induced disruption of gastrointestinal integrity

**DOI:** 10.1007/s00421-020-04382-w

**Published:** 2020-05-12

**Authors:** F. C. Mooren, B. H. Maleki, C. Pilat, R. Ringseis, K. Eder, M. Teschler, K. Krüger

**Affiliations:** 1 Faculty of Health, School of Medicine, Witten/Herdecke University, Witten, Germany; 2 Department of Exercise Physiology and Sports Therapy, Institute of Sports Sciences, Justus-Liebig-University, Giessen, Germany; 3 Institute of Animal Nutrition and Nutrition Physiology, Justus-Liebig-University, Giessen, Germany

**Keywords:** *Escherichia coli* strain Nissle 1917, Exercise, Gastrointestinal tract, Probiotic, Intestinal fatty acid binding protein

## Abstract

**Purpose:**

The aim of the current study was to investigate the effects of the probiotic *Escherichia coli* strain Nissle 1917 (EcN) on the exercise-induced disruption of gastrointestinal (GI) integrity and the associated release of damage and inflammatory markers.

**Methods:**

After a pre-performance test, 19 untrained subjects (aged 18–35 years) passed two identical exhaustive treadmill exercise tests in an intensity corresponding to 60–80% *V*O_2max_ in a test–retest design. The exercise tests were separated by a time period of 4 weeks. During this period, all subjects ingested 5 ml of an EcN suspension daily. Serum samples were taken before, immediately following and 3 h after both exercise tests. They were analyzed for indicators of GI integrity (zonulin; claudin-3; LPS), various damage and redox markers (I-FABP, GOT; GPT; TBARS) and inflammatory parameters (hsCRP; leucocytes). GI complaints were evaluated by a questionnaire.

**Results:**

The intake of EcN resulted in a significantly lower increase in I-FABP and TBARS after exercise (*p* < 0.05). In contrast, no effect of EcN supplementation was found for hsCRP and leucocyte numbers. Similarly, no differences were found for levels of zonulin and claudin-3. Exercise-associated GI complaints were not affected by the probiotic supplement.

**Conclusion:**

The probiotic EcN reduced the exercise-associated increase in oxidative stress. This antioxidative mechanism probably leads to a reduction of GI epithelial damage after exhaustive exercise. The lack of EcN effects on other markers of GI permeability and systemic inflammation is most likely due to an inadequate exercise load, with rather small and insignificant exercise effects on these parameters.

## Introduction

A considerable number of athletes in recreational and competitive sports regularly report exercise-associated complaints of the gastrointestinal (GI) tract. These range from nausea, vomiting, meteorism, reflux syndrome, intestinal cramps, and diarrhea up to hematochezia. Especially endurance athletes report high incidence rates for GI symptoms of up to 50% (Riddoch and Trinick 1988). Thereby, symptoms of the lower GI tract seem to be more prevalent in runners, while cyclists report symptoms of both upper and lower GI tract similarly (Peters et al. 1999). Moreover, the exercise-associated GI dysfunction may have considerable consequences for exercise performance. About a third of those affected by such GI dysfunctions describe that exercise performance is significantly impaired (Halvorsen and Ritland 1992).

GI dysfunction is assumed to be a result of changes in motility, absorption, and secretory processes, which are affected by stress and brain–gut interactions as well as mechanical factors. Another important pathogenetic step is a reduced GI blood flow during exercise, followed by a reperfusion after exercise (Konturek et al. 2009; van Wijck et al. 2012). The exercise-induced perturbations of perfusion, away from the GI tract toward muscle and skin, are a challenge predominantly for untrained and unexperienced athletes (Mooren and Stein 2011). The resulting changes of perfusion, such as hypoxia, hyperthermia, and reoxygenation, lead to changes in redox balance. This affects the cell/tissue structure and local metabolism massively, followed by a loss of GI integrity (van Wijck et al. 2012; Gutekunst et al. 2014). Electrolyte transport and tissue conductivity are increased during and after exercise, indicating an increased permeability and tight junction disorder (Mooren and Stein 2011). The increased tissue permeability is followed by an influx of lipopolysaccharides (LPS) from the intestine into the circulation. This is then followed by a slight systemic proinflammatory response (Bosenberg et al. 1988; Jeukendrup et al. 2000).

Regarding therapeutic options, predominantly symptomatically effective compounds such as antacids or proton pump inhibitors are frequently prescribed. In case of severe symptoms, athletes are advised to change both training type and frequency. However, such changes in the training regime are not well accepted by most athletes. Therefore, alternative therapy options would be preferable (Mooren and Stein 2011).

There is evidence that probiotics may serve as an effective and safe approach for both the prevention and treatment of exercise-associated GI dysfunction (Pugh et al. 2019). Moreover, probiotics have been shown to enhance exercise performance (Shing et al. 2014). However, there is limited evidence about the underlying mechanisms so far.

Therefore, the aim of the current study was to investigate whether the probiotic medication *Escherichia coli* strain Nissle 1917 (EcN) can effectively mitigate the effect of exhausting exercise on GI damage and permeability.

## Methods

### Participants

Twenty untrained (*V*O_peak_ < 53 ml/kg min) male volunteers (aged 18–35 years) were recruited as participants (Table 1). One subject was excluded because he did not complete both exercise trials, due to personal reasons. All participants were in good health, as determined by a regular physical examination and routine laboratory tests. They were non-smokers. None of them were taking any supplements or dieting, i.e., restricting their caloric intake for the sake of weight loss. The experimental procedures and potential risks were explained to all subjects before their written informed consent was obtained. The study was approved by the Ethics Committee of the Justus-Liebig-University, Giessen, Germany.

## Experimental design

**Figure 1:**
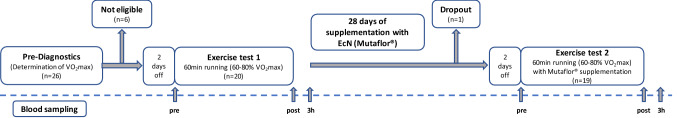
Study design overview

The trial consisted of 3 days of experiments (Fig. 1). On the first day, after a physical examination, participants performed a progressive exercise test to exhaustion, for the determination of *V*O_2peak_ and sports eligibility. After a break of at least 3 days and an exercise free period of at least 48 h, subjects performed a strenuous exercise test (test 1; day 2) on the treadmill for 1 h with increasing intensity, according to the following progression: 10 min warm-up at an intensity corresponding to 60% *V*O_2peak_, 25 min corresponding to 70% *V*O_2peak_, and 25 min corresponding to 80% *V*O_2_max or until exhaustion, respectively.

After finishing test 1, participants received an ampulla containing 5 ml suspension of EcN (Mutaflor^®^), which was applied daily for a period of 28 days. After this period, a second, identical (with respect to intensity and duration) strenuous exercise test was performed (test 2; day 3). Again, subjects had to refrain from exercise for at least 48 h before the exercise test. The test’s retest design was used because we could not exclude that EcN settles in the intestinal flora (Joeres-Nguyen-Xua et al. 2010).

Initial blood samples were taken at rest before exercise. Additional samples were collected immediately and 3 h after both exercise trials.

### Gastrointestinal symptoms and physical activity

Gastrointestinal symptoms during and up to 3 h after the run were determined using a self-developed questionnaire covering 19 items, such as stomach cramps, nausea and heartburn, on a ten-point Likert scale and an overall GI symptomatology rating on a visual analog scale. Physical activity was determined using the IPAQ questionnaire, to control the subjects’ activity levels during the last 7 days before the exercise trials (Craig et al. 2003).

### Blood sampling

Venous blood samples were obtained through venipuncture from the median vein and collected in plastic tubes, anticoagulated with either ethylenediaminetetraacetate (EDTA) for blood cell count or without anticoagulants for serum generation (S-Monovette 7.5ml and EDTA Monovette, Sarstedt AG & Co. KG, Germany). The serum samples were centrifuged at 2000*g* for 15 min and then stored in aliquots at – 80 °C until analysis. Complete blood cell counts were determined using a Sysmex automated cell counter (Sysmex Deutschland GmbH, Germany).

### Biochemical analysis

Levels of serum LPS, zonulin, intestinal fatty acid-binding protein (I-FABP), claudin-3 (CLDN3), high-sensitive C-reactive protein (hsCRP) were determined using enzyme-linked immunosorbent assay (ELISA) kits from R&B systems, USA. The serum activity of aspartate aminotransferase (GOT) and alanine aminotransferase (GPT) was measured using a fluorometric assay. Plasma concentration of thiobarbituric acid reactive substances (TBARS) was determined spectrofluorimetrically. Briefly, plasma samples were heated with thiobarbituric acid reagent at 100 °C for 60 min. After cooling, it was neutralized with alkaline methanol. Finally, samples were centrifuged at 3000 × *g*. TBARS levels were measured by fluorescence signals (excitation wavelength, 532 nm; emission wavelength, 553 nm; Fluorescence Spectrometer LS55, PerkinElmer, Rodgau, Germany).

### Statistical analysis

All statistical analyses were carried out with SPSS version 24 (IBM^®^ SPSS Statistics 24, IBM GmbH, München, Germany). Depending on their distribution, all data were presented either as arithmetic mean ± standard deviation or median ± interquartile range. The primary efficacy end points of the study were defined as the score of the area under curve, with respect to the increase (AUCI) of LPS and zonulin, over a period of 3 h post-exercise. The calculation of the AUCI score was performed using the trapezoidal rule, according to Pruessner et al. (2003). The AUCI is defined as the area under the curve above the baseline level, minus the area above the curve below the baseline level. It is a measure of the pattern of change of repeated measurements and considers differences between the time intervals (Pruessner et al. 2003). For analysis of treatment effects, a Student’s *t* test or a non-parametric Wilcoxon test of the AUCI score was performed according to the intention-to-treat (ITT) principle. Significance was set at the one-sided *p* < 0.05 level. All secondary outcome parameters were tested accordingly to two-sided tests. Additionally, as part of the exploratory data analysis, a univariate analysis of variance (ANOVA) with the factor time point was performed for all outcome parameters. Both exercise tests were subsequently corrected during post hoc analysis using the Bonferroni–Holm method.

## Results

The anthropometric data of the study participants are presented in Table 1. With an average maximum oxygen uptake of about 46 ml O_2_/min/kg of body weight, the sample showed the anticipated low performance level. The level of physical activity before the two performance tests revealed no significant difference (data not shown).

Regarding the intake of the study medication, the participants’ compliance can be described as satisfactory. 13 out of 19 study participants (68%) took the study medication according to the protocol. Of the remaining six, two participants each (1%) forgot to take either one, two or three ampoules, respectively, during the supplementation period. In summary, 97.7% of the intended dose of EcN was administered. Heart rate recordings during both protocols and the comparison of blood counts showed that the exercise protocols were almost identical regarding intensity and duration.

**Figure 2:**
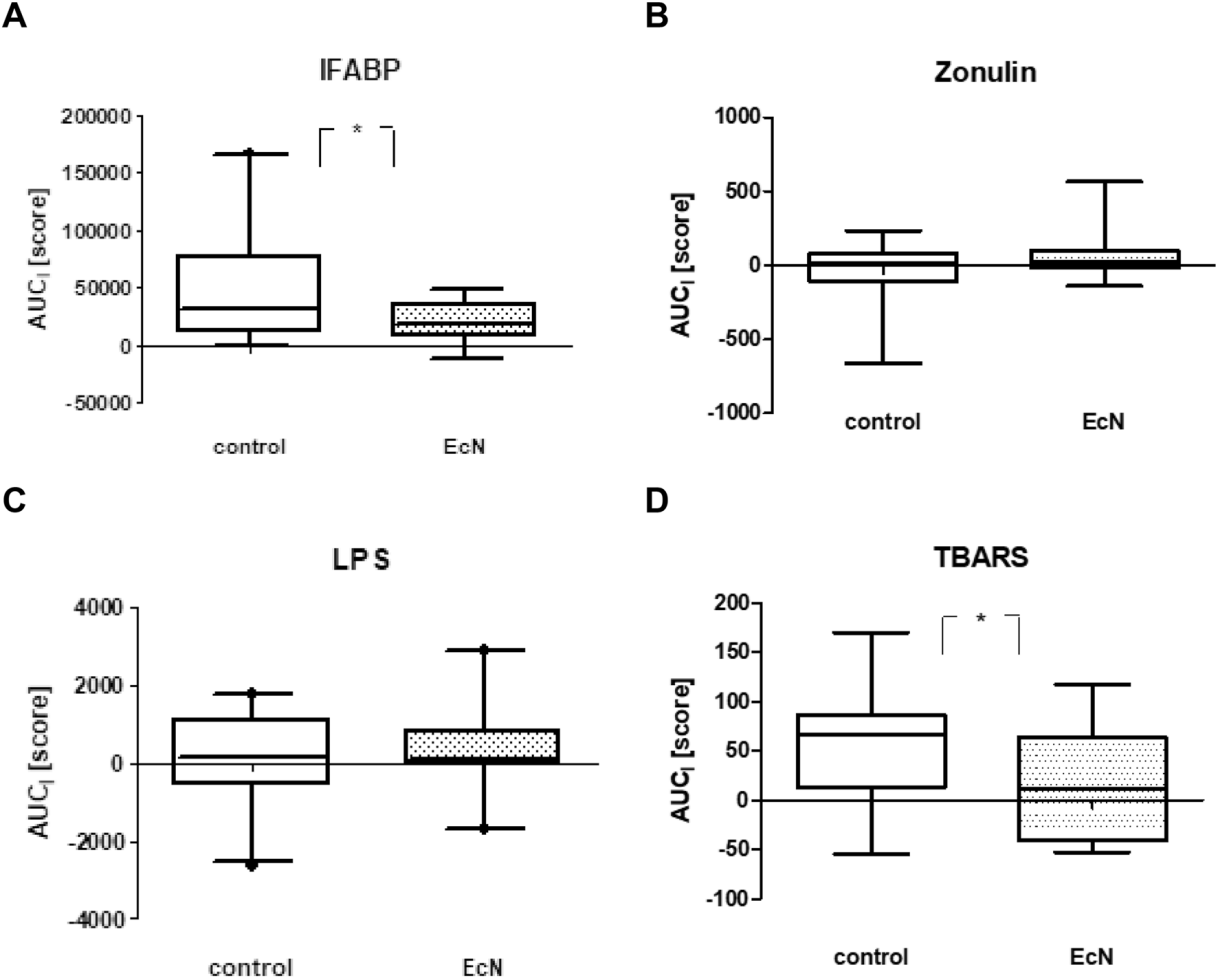
AUC_I_ score of exercise-induced I-FABP (**a**) and TBARS (**b**) without medication (control) or a preceding 4-week Mutaflor^®^-intake (EcN). Arithmetic means and 95th percentiles are given. *p < 0.05

Exhaustive exercise resulted in a highly significant increase of the intestinal damage marker I-FABP in both groups. In comparison, the EcN group showed a significantly lower increase in I-FABP than the control group (Fig. 2a; Table 2). There were no consistent effects of exercise on gut integrity markers such as zonulin, CLDN3, and LPS. Likewise, we could not find any EcN treatment effects on these parameters (see Fig. 2b, c). The generation of reactive oxygen species as indicated by the formation of TBARS was significantly enhanced after both exercise bouts. Moreover, there was a substantial treatment effect after EcN supplementation, i.e., lower TBARS levels ensued (Fig. 2d).

The liver and muscle damage markers GPT and GOT were slightly affected by exercise. However, there was no difference between treatment groups. Similar effects were found for systemic inflammation parameters. Finally, following both treatment conditions, GI symptoms did not show any significant differences (data not shown).

## Discussion

An essential result of the study is that exercise stress-induced damage of the GI epithelium can be partly reduced by a prophylactic administration of EcN. Regarding the underlying mechanism, a significantly lower oxidative stress suggests an antioxidative effect of the probiotic medication. The assumption of a lower oxidative stress is based on the observation that serum TBARS levels have been reduced by the probiotic medication. Despite this, TBARS are generally considered critical due to low specificity, i.e., TBARS react with various substrates to form malondialdehyde (MDA). Mostly, MDA is generated by the assay itself. The wide acceptance and frequent utilization of this organic compound in biomedical research can be explained thus: it reliably reflects the sensitivity of any tissues toward oxidation and therefore has a positive effect, even though there are unspecific indicators of oxidative stress. It cannot be ruled out that serum TBARS might, to some extent, reflect oxidative stress in erythrocytes. Still, gut microbiota-induced intestinal injury was reported to cause systemic oxidative stress in the host. This became evident from increased serum levels of TBARS and other oxidative stress-related parameters (Wang et al. 2018). However, there are other plausible direct and indirect mechanisms of microbiota modulation that affect the gut barrier function. Among these is the increasing expression of intestinal antimicrobial peptides, which reduces bacterial overgrowth and stimulates mucosal immunity (Gotteland et al. 2001).

Exercise can negatively affect the integrity of the GI epithelium. This ranges from simple apoptotic cell death over a tight junction opening and an associated increase in GI permeability, up to pronounced hemorrhagic diarrhea with a colonoscopy picture similar to ischemic colitis (Halvorsen and Ritland 1992; Riddoch and Trinick 1988). Important influencing factors are the exercise intensity, volume and type of exercise, the athlete’s training status as well as the quality and quantity of food and fluid intake (Mooren and Stein 2011). GI complaints are characterized by a variety of symptoms, such as flatulence, abdominal cramps, nausea, diarrhoea and the urge to stool (Peters et al. 1999; Riddoch and Trinick 1988). Comparable to our findings, no significant correlation between the extent of the GI damage, as indicated by endotoxemia and clinical symptoms, could be demonstrated so far (Jeukendrup et al. 2000). An important causal role in triggering exercise-induced GI dysfunction is attributed to the reduced blood flow and resulting reoxygenation of the GI tract (van Wijck et al. 2012). These processes disturb tissue homeostasis by conditions of hypoxia, hyperthermia, and an increased production of free radicals. The mucosal barrier is weakened by the disruption of tight and adherent junctions between the enterocytes. An important modulator of intestinal tight junctions is zonulin. Its detachment from the protein complex of tight junctions leads to an opening of the paracellular pathway and an associated increase in GI permeability. Thus, zonulin is regarded as an important marker for the integrity of the intestinal mucosa (Fasano 2012). The disturbance of GI permeability leads to an influx of LPS into the circulation, thereby initiating subsequent proinflammatory reactions (Bosenberg et al. 1988; Jeukendrup et al. 2000). Since no significant changes of these markers were found, it is suggested that exercise intensity and/or duration was below the threshold to achieve a more extensive damage. Thus, they failed to influence GI integrity and permeability. Likewise, Karhu et al. showed no serum LPS changes after a 90-min run at 80% of the maximum/best 10 km pace (Karhu et al. 2017). This assumption is also supported by the lack of any gastrointestinal complaints. It is assumed that due to these findings, no effect of EcN application was found.

In contrast, we detected a significant exercise-induced increase of I-FABP in both groups, suggesting that I-FABP may be an extremely sensitive marker of intestinal cell damage. Such a conclusion is supported by animal experiments showing serum I-FABP increases even after a short period of ischemia. It can therefore be accepted as a highly sensitive and early marker for GI damage (Kanda et al. 1992; Niewold et al. 2004).

To our knowledge, this is the first study to demonstrate that a prophylactic use of EcN can reduce the extent of exercise-induced GI damage in humans. Previous studies provided evidence of improved running performance in both laboratory and field exercise test after 4 weeks of probiotic use (Pugh et al. 2019; Shing et al. 2014). However, they failed to demonstrate any effects on biochemical and inflammatory parameters. Merely animal experiments demonstrated that probiotics reduced intestinal damage. Using fermented milk supplemented with whey protein, probiotic and pomegranate, Chaves et al. (2018) could demonstrate a preserved intestinal motility and villi structure in exercised rats. In contrast, physical performance was not improved by fermented milk supplementation.

From a clinical point of view, there is some evidence that EcN may be beneficial in the treatment of some chronic intestinal ailments and that it can at least limit tissue damage caused by inflammatory intestinal diseases (Petrof 2009). In humans, EcN is used for the treatment of ulcerative colitis (Jia et al. 2018). EcN is also used to treat functional intestinal disorders such as irritable bowel syndrome. These clinical data fit with those provided by Pugh et al. (2019), which showed significantly fewer GI symptoms after probiotic administration in marathon runners, compared to the control group. Accordingly, these data are an additional hint that the exercise protocol performed in the current study was not enough to induce pronounced GI complaints.

The current study addresses some possible working mechanisms how EcN may protect the GI epithelia. As indicated by differences in the TBARS serum level, a widely accepted and well-known indicator of tissue sensitivity regarding oxidation, it can be assumed that EcN supplementation affects the redox balance during exercise. This effect on redox balance, while well known from other probiotics, has not yet been described for probiotic EcN. In this regard, animal studies give evidence for an antiapoptotic effect of EcN on enterocytes that are incubated with a cell toxin (Prisciandaro et al. 2012). Within such an experimental setup, free radicals play an important triggering role, even though they were not investigated in this study (Prisciandaro et al. 2012).

Several other studies showed an anti-inflammatory effect of EcN (Sha et al. 2014; Souza et al. 2016). Using a combined physiological and psychological stress model, Konturek et al. (2009) were able to describe a significant downregulation of mRNA and protein level for IL-1, Cox-2 and PPAR-gamma, as well as an increased expression of the heat shock protein Hsp70. The reduced formation of stress ulcers after EcN supplementation was associated with an increased gastric blood flow (Konturek et al. 2009). The decreased GI blood flow during intense exercise is considered to be a significant trigger of functional and morphological changes in the intestinal epithelium and an immune system activator. Transient ischemia as well as the subsequent hyperemia are associated with an increased accumulation of oxidatively active substances, such as free radicals (van Wijck et al. 2012). The antioxidative properties of probiotics have generally been known for a long time (Coskun Cevher et al. 2015; Pieniz et al. 2015). Various factors are probably responsible for this feature, e.g., generation of radical scavenging substances and induction of radical depleting enzymes in host tissues (Wang et al. 2017).

A limited number of studies have indicated that the bacterial modulation of the microbiota by probiotics affects gut barrier integrity in (healthy) human volunteers. In this regard, Gotteland et al. (2001) demonstrated that the consumption of live *Lactobacillus rhamnosus* protected the integrity of the gastric mucosal barrier. However, an effect on the intestinal barrier was not found.

Finally, some limitations must be addressed. It was unexpected that, following both treatment conditions, GI symptoms did not show any significant differences. It is assumed that the exercise trials were not sufficient to induce complaints connected to the gastrointestinal (GI) tract. Since EcN has the ability to settle in the intestinal flora of its host, a crossover design could not be applied in the present trial (Joeres-Nguyen-Xuan et al. 2010). After termination of EcN supplementation, it is hardly possible to define a certain washout phase, making it difficult to guarantee an EcN-free microbiome over a couple of months. Therefore, a crossover study would not be feasible within a reasonable time frame, a circumstance that forced the researchers to choose the present test–retest design. Consequently, it should be mentioned that it is difficult to eliminate any confounding period effects, e.g., a GI habituation to the exercise stimulus. The authors feel that this effect can be neglected in particular after a 4-week washout phase.

## Conclusion

The present study shows that a 4-week administration of EcN can significantly reduce exercise-induced GI damage in untrained athletes. Here, the influence of EcN on redox balance seems to play an important causal role. However, since no changes in GI complaints were found, the therapeutic and clinical relevance of these results remains unclear. Further studies are necessary to investigate the effects of EcN on GI complaints and exercise performance under conditions of a higher exercise load, to further evaluate its potential benefits in more detail.

**Table 1:** Anthropometric data and basal characteristics of study participants (*n* = 19; mean ± SD)

Characteristics	Value
Age	26.5 ± 4.7
Weight (kg)	75.1 ± 7.3
Height (cm)	180.0 ± 5.5
BMI (kg/m^2^)	23.2 ± 1.9
*V*O_2max_	46.0 ± 3.6

**Table 2:** Arithmetic mean ± standard deviation of every outcome measure before (pre), immediately after (post), 3 h after exercise (3 h)

Outcomes measure		Time point							
Gut integrity		Pre	Post		3h		ANOVA time effect (*p* value)	AUC_i_ (score)	Treatment effect (*p* value)
Zonulin (ng/ml)	con	3.00 ± 2.35	3.03 ± 2.70		2.76 ± 2.20		0.345	− 28.6 ± 210.1	0.084
	M	2.40 ± 2.31	2.90 ± 2.37		2.42 ± 2.28		**0.016**^**ac**^	75.3 ± 195.2	
LPS (pg/ml)	con	34:5 ± 19.8	36.8 ± 16.7		37.0 ± 19.0		0.659	197.2 ± 1063.4	0.362
	M	30.4 ± 17.0	34.5 ± 16.9		31.0 ± 17.5		**0.047**^**c**^	324.2 ± 1133.1	
CLDN3 (pg/ml)	con	65.9 ± 14.3	71.9 ± 11.4		75.3 ± 9.6		0.239	743.5 ± 1967.4	0.844
	M	63.8 ± 15.2	73.3 ± 20.9		63.8 ± 16.5		**0.019**^**a**^	859.5 ± 2030.0	
Redox status									
TBARS (µmol/l)	con	2.1 ± 0.6	2.5 ± 0.4		2.3 ± 0.4		**0.001**^**a**^	55.9 ± 15.3	**0.025**
	M	2.2 ± 0.4	2.4 ± 0.3		2.3 ± 0.3		**0.001**^**c**^	18.8 ± 14.6	
Intestinal damage									
I-FABP (pg/ml)	con	384.3 ± 450.9	559.8 ± 465.6		474.7 ± 491.6		**0.000**^**ab**^	45682.0 ± 50829.3	**0.037**
	M	390.6 ± 524.4	509.4 ± 456.7		552.1 ± 503.5		**0.003**^**ab**^	20305.8 ± 17478.7	
Liver/muscle damage									
GPT (U/l)	con	32.4 ± 7.5	34.4 ± 8.8		31.7 ± 6.9		**0.000**^**ab**^	78.9 ± 294.3	0.559
	M	46.7 ± 36.1	47.9 ± 35.6		46.0 ± 33.3		0.153	129.5 ± 340.2	
GOT (U/l)	con	32.4 ± 13.8	35.4 ± 13.4		35.6 ± 13.0		**0.000**^**ab**^	612.6 ± 363.8	0.496
	M	40.3 ± 21.0	44.0 ± 22.6		42.0 ± 19.0		**0.000**^**ab**^	682.1 ± 301.0	
Systemic, inflammatory status									
hsCRP (mg/l)	con	0.6 ± 0.7	0.6 ± 0.7		0.6 ± 0.7		0.143	3.5 ± 8.3	0.248
	M	1.6 ± 2.9	1.7 ± 3.1		1.8 ± 3.4		**0.047**	111.3 ± 294.0	
Leukocytes (× 10^6^/ml)	con	5.2 ± 1.0	6.7 ± 1.5		9.5 ± 2.5		**0.000**^**abc**^	578.2 ± 274.4	0.703
	M	5.5 ± 1.3	7.3 ± 1.5		9.7 ± 1.5		**0.000**^**abc**^	552.0 ± 282.7	
Red blood cell count and indices									
Erythrocytes (× 10^6^/µl)	con	5.0 ± 0.3	5.1 ± 0.3		5.0 ± 0.3		**0.000**^**ac**^	20.4 ± 27.0	0.676
	M	4.9 ± 0.3	5.1 ± 0.3		5.0 ± 0.3		**0.000**^**ac**^	24.1 ± 28.2	
Hematocrit (%)	con	44.2 ± 2.9	45.1 ± 2.8		43.6 ± 3.3		**0.001**^**ac**^	60.3 ± 253.6	0.612
	M	44.9 ± 2.4	46.0 ± 2.8		44.3 ± 3.1		**0.001**^**ac**^	102.5 ± 267.2	
Hemoglobin (g/dl)	con	15.0 ± 1.0	15.4 ± 1.0		15.0 ± 1.1		**0.000**^**ac**^	56.8 ± 78.3	0.815
	M	15.0 ± 0.9	15.5 ± 1.0		15.0 ± 1.0		**0.001**^**ac**^	63.5 ± 110.3	
Thrombocytes (× 10^3^/µl)	con	218 ± 40	277 ± 49		231 ± 44		**0.000**^**abc**^	8445.8 ± 4024.1	0.987
	M	214 ± 36	278 ± 53		224 ± 38		**0.000**^**abc**^	8461.6 ± 3981.2	
MCV (fl)	con	88.7 ± 3.1	87.6 ± 3.0		87.5 ± 3.0		**0.000**^**ab**^	− 232.3 ± 102.5	0.936
	M	90.9 ± 3.4	89.9 ± 3.5		89.5 ± 3.1		**0.000**^**ab**^	− 234.8 ± 161.9	
MCH (pg/Ery)	con	30.2 ± 1.1	30.2 ± 1.1		30.2 ± 1.1		0.765	− 5,8 ± 69.0	0.568
	M	30.3 ± 1.0	30.2 ± 1.1		30.3 ± 1.0		0.591	− 16.3 ± 74.2	
MCHC (g/dl Ery)	con	34.0 ± 0.9	34.3 ± 0.8		34.4 ± 0.8		**0.002**^**ab**^	77.7 ± 80.4	0.756
	M	33.4 ± 0.9	33.7 ± 0.9		33.8 ± 0.8		**0.003**^**ab**^	71.4 ± 93.8	

The respective AUC_I_ scores of the control condition (con) and with prior Mutaflor^®^-intake (M), *p* values for ANOVA time effects (a: pre vs. post, b: pre vs. 3 h, c: post vs. 3 h), and AUC_I_ comparisons are given

Bold values show significant changes

## Abbreviations

GI: Gastrointestinal
LPS: Lipopolysaccharides
EcN: *Escherichia coli* Strain Nissle 1917
*V*O_2max_: Maximal oxygen consumption
IPAQ: International Physical Activity Questionnaire
EDTA: Ethylenediaminetetraacetate
I-FABP: Intestinal fatty acid-binding protein
TBARS: Thiobarbituric acid reactive substances
hsCRP: High-sensitivity C-reactive protein
ELISA: Enzyme-linked immunosorbent assay
GOT: Aspartate aminotransferase
GPT: Alanine aminotransferase
AUCI: Area under curve
CLDN3: Claudin 3 gene
mRNA: Messenger-RNA
IL-1: Interleukin-1-beta
COX-2: Cyclooxygenase-2
PPAR-gamma: Peroxisome proliferator-activated receptor gamma

## References

[CR1] Bosenberg AT, Brock-Utne JG, Gaffin SL, Wells MT, Blake GT (1988) Strenuous exercise causes systemic endotoxemia. J Appl Physiol (1985) 65(1):106–108. https://doi.org/10.1152/jappl.1988.65.1.106 10.1152/jappl.1988.65.1.106 3403455

[CR2] Chaves FM, Baptista IL, Simabuco FM, Quaresma PGF, Pena FL, Bezerra RMN, Pauli JR, da Cunha DT, Campos-Ferraz PL, Antunes AEC (2018) High-intensity-exercise-induced intestinal damage is protected by fermented milk supplemented with whey protein, probiotic and pomegranate (*Punica granatum* L.). Br J Nutr 119 (8):896–909. 10.1017/S0007114518000594 10.1017/S0007114518000594 29644961

[CR3] Coskun Cevher S, Balabanli B, Aslim B (2015). Effects of probiotic supplementation on systemic and intestinal oxidant-antioxidant events in splenectomized rats. Surg Today.

[CR4] Craig CL, Marshall AL, Sjostrom M, Bauman AE, Booth ML, Ainsworth BE, Pratt M, Ekelund U, Yngve A, Sallis JF, Oja P (2003). International physical activity questionnaire: 12-country reliability and validity. Med Sci Sports Exerc.

[CR5] Fasano A (2012). Intestinal permeability and its regulation by zonulin: diagnostic and therapeutic implications. Clin Gastroenterol Hepatol.

[CR6] Gutekunst K, Kruger K, August C, Diener M, Mooren FC (2014). Acute exercises induce disorders of the gastrointestinal integrity in a murine model. Eur J Appl Physiol.

[CR7] Gotteland M, Cruchet S, Verbeke S (2001). Effect of Lactobacillus ingestion on the gastrointestinal mucosal barrier alterations induced by indometacin in humans. Aliment Pharmacol Ther.

[CR8] Halvorsen FA, Ritland S (1992). Gastrointestinal problems related to endurance event training. Sports Med.

[CR9] Jeukendrup AE, Vet-Joop K, Sturk A, Stegen JH, Senden J, Saris WH, Wagenmakers AJ (2000) Relationship between gastro-intestinal complaints and endotoxaemia, cytokine release and the acute-phase reaction during and after a long-distance triathlon in highly trained men. Clin Sci (Lond) 98 (1):47–55 10600658

[CR10] Jia K, Tong X, Wang R, Song X (2018). The clinical effects of probiotics for inflammatory bowel disease: a meta-analysis. Medicine (Baltimore).

[CR11] Joeres-Nguyen-Xuan TH, Boehm SK, Joeres L, Schulze J, Kruis W (2010). Survival of the probiotic *Escherichia coli* Nissle 1917 (EcN) in the gastrointestinal tract given in combination with oral mesalamine to healthy volunteers. Inflamm Bowel Dis.

[CR12] Kanda T, Nakatomi Y, Ishikawa H, Hitomi M, Matsubara Y, Ono T, Muto T (1992). Intestinal fatty acid-binding protein as a sensitive marker of intestinal ischemia. Dig Dis Sci.

[CR13] Karhu E, Forsgard RA, Alanko L, Alfthan H, Pussinen P, Hamalainen E, Korpela R (2017). Exercise and gastrointestinal symptoms: running-induced changes in intestinal permeability and markers of gastrointestinal function in asymptomatic and symptomatic runners. Eur J Appl Physiol.

[CR14] Konturek PC, Sliwowski Z, Koziel J, Ptak-Belowska A, Burnat G, Brzozowski T, Konturek SJ (2009). Probiotic bacteria *Escherichia coli* strain Nissle 1917 attenuates acute gastric lesions induced by stress. J Physiol Pharmacol.

[CR15] Mooren FC, Stein B (2011). Potentially detrimental effects of marathon on the gastrointestinal system. D Z Sportmed.

[CR16] Niewold TA, Meinen M, van der Meulen J (2004). Plasma intestinal fatty acid binding protein (I-FABP) concentrations increase following intestinal ischemia in pigs. Res Vet Sci.

[CR17] Peters HP, Bos M, Seebregts L, Akkermans LM, van Berge Henegouwen GP, Bol E, Mosterd WL, de Vries WR (1999). Gastrointestinal symptoms in long-distance runners, cyclists, and triathletes: prevalence, medication, and etiology. Am J Gastroenterol.

[CR18] Petrof EO (2009). Probiotics and gastrointestinal disease: clinical evidence and basic science. Antiinflamm Antiallergy Agents Med Chem.

[CR19] Pieniz S, Andreazza R, Okeke BC, Camargo FA, Brandelli A (2015). Antimicrobial and antioxidant activities of Enterococcus species isolated from meat and dairy products. Braz J Biol.

[CR20] Prisciandaro LD, Geier MS, Chua AE, Butler RN, Cummins AG, Sander GR, Howarth GS (2012). Probiotic factors partially prevent changes to caspases 3 and 7 activation and transepithelial electrical resistance in a model of 5-fluorouracil-induced epithelial cell damage. Support Care Cancer.

[CR21] Pruessner JC, Kirschbaum C, Meinlschmid G, Hellhammer DH (2003). Two formulas for computation of the area under the curve represent measures of total hormone concentration versus time-dependent change. Psychoneuroendocrinology.

[CR22] Pugh JN, Sparks AS, Doran DA, Fleming SC, Langan-Evans C, Kirk B, Fearn R, Morton JP, Close GL (2019). Four weeks of probiotic supplementation reduces GI symptoms during a marathon race. Eur J Appl Physiol.

[CR23] Riddoch C, Trinick T (1988). Gastrointestinal disturbances in marathon runners. Br J Sports Med.

[CR24] Sha S, Xu B, Kong X, Wei N, Liu J, Wu K (2014). Preventive effects of *Escherichia coli* strain Nissle 1917 with different courses and different doses on intestinal inflammation in murine model of colitis. Inflamm Res.

[CR25] Shing CM, Peake JM, Lim CL, Briskey D, Walsh NP, Fortes MB, Ahuja KD, Vitetta L (2014). Effects of probiotics supplementation on gastrointestinal permeability, inflammation and exercise performance in the heat. Eur J Appl Physiol.

[CR26] Souza EL, Elian SD, Paula LM, Garcia CC, Vieira AT, Teixeira MM, Arantes RM, Nicoli JR, Martins FS (2016). *Escherichia coli* strain Nissle 1917 ameliorates experimental colitis by modulating intestinal permeability, the inflammatory response and clinical signs in a faecal transplantation model. J Med Microbiol.

[CR27] van Wijck K, Lenaerts K, Grootjans J, Wijnands KA, Poeze M, van Loon LJ, Dejong CH, Buurman WA (2012). Physiology and pathophysiology of splanchnic hypoperfusion and intestinal injury during exercise: strategies for evaluation and prevention. Am J Physiol Gastrointest Liver Physiol.

[CR28] Wang Y, Wu Y, Wang Y, Xu H, Mei X, Yu D, Wang Y, Li W (2017) Antioxidant properties of probiotic bacteria. Nutrients 9(5). 10.3390/nu9050521 10.3390/nu9050521 PMC5452251 28534820

[CR29] Wang H, Ji Y, Yin C, Deng M, Tang T, Deng B, Ren W, Deng J, Yin Y, Tan C (2018). Differential analysis of Gut microbiota correlated with oxidative stress in sows with high or low litter performance during lactation. Front Microbiol.

